# A segmental radiological study of the spine and rib – cage in children with progressive Infantile Idiopathic Scoliosis

**DOI:** 10.1186/1748-7161-1-17

**Published:** 2006-10-18

**Authors:** Theodoros B Grivas, Geoffrey R Burwell, Elias S Vasiliadis, John K Webb

**Affiliations:** 1 Orthopaedic Department, "Thriasion" General Hospital, G. Gennimata Av. 19600, Magoula, Attica, Greece; 2 School of Biomedical Sciences, Medical School, Queen's Medical Centre, University of Nottingham, Nottingham, NG7 2UH, UK; 3 Centre for Spinal Studies, University Hospital, Nottingham, NG7 2UH, UK

## Abstract

**Background:**

The role of rib cage in the development of progressive infantile idiopathic scoliosis (IIS) has not been studied previously. No report was found for rib growth in children with IIS. These findings caused us to undertake a segmental radiological study of the spine and rib-cage in children with progressive IIS. The aim of the present study is to present a new method for assessing the thoracic shape in scoliotics and in control subjects and to compare the findings between the two groups.

**Materials and methods:**

In the posteroanterior (PA) spinal radiographs of 24 patients with progressive IIS, with a mean age of 4.1 years old, the Thoracic Ratios (TRs) (segmental convex and concave TRs), the Cobb angle, the segmental vertebral rotation and vertebral tilt were measured. In 233 subjects, with a mean age of 5.1 years old, who were used as a control group, the segmental left and right TRs and the total width of the chest (left plus right TRs) were measured in PA chest radiographs. Statistical analysis included Mann-Whitney, Spearman correlation coefficient, multiple linear regression analysis and ANOVA.

**Results:**

The comparison shows that the scoliotic thorax is significantly narrower than that of the controls at all spinal levels. The upper chest in IIS is funnel-shaped and the vertebral rotation at T4 early in management correlates significantly with the apical vertebral rotation at follow up.

**Conclusion:**

The IIS thorax is narrower than that of the controls, the upper chest is funnel-shaped and there is a predictive value of vertebral rotation at the upper limit of the thoracic curve of IIS, which reflects, impaired rib control of spinal rotation possibly due to neuromuscular factors, which contribute also to the funnel-shaped chest.

## Background

A study of children aged 1–5 years with progressive infantile idiopathic scoliosis (IIS) shows that a two stage anterior and posterior surgical procedure leads to deterioration of the spine during follow up [1-3].

By reviewing the literature, no publication was found on rib growth in children with IIS. Dansereau et al [4] examined rib length asymmetry by using a stereoradiographic method in children with adolescent idiopathic scoliosis.

These findings caused us to undertake a segmental radiological study of the spine and rib – cage in children with progressive IIS.

The effect of upper thoracic cage on pulmonary function in IIS is an additional interesting issue. A report on diminished pulmonary function following arthrodesis of the thoracic spine before age 5 noted strong correlation with early age and number of thoracic vertebrae fused in a group of patients without pre-existing chest wall or neuromuscular disease [5]. While progression of deformity necessitates surgical stabilization, fusion by default limits the capability of the thoracic cavity to grow normally [6,7]. If the chest cannot elongate with growth, there is insufficient space available for pulmonary alveolar growth, with resultant extrinsic restrictive lung disease [8-10].

Reference standards are available for chest shape, chest volume, spine length, and segmental spine growth by age [11,12] but are based on normals rather than IIS patients with altered skeletal proportions and altered rates of growth [13]. This study reports reference values of chest dimensions in growing individuals, which are less dependent on normal growth rate, body proportion, and stature and which will be useful in assessing the effect of treatment of IIS.

The aim of the present study is to present a new method for assessing the thoracic shape in scoliotics with IIS and in control subjects and to compare the findings between the two groups.

## Methods and materials

### Measurement of chest radiographs. The Thoracic Ratios

**Figure 1:**
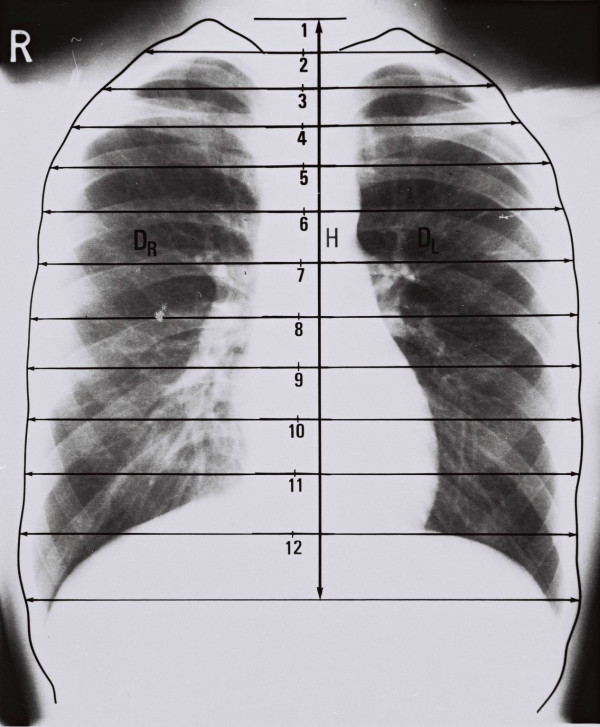
The method of thoracic ratios. Chest radiograph to show the method of measurement of calculation of thoracic ratios (TRs). **H **represents the distance from the upper end-plate of the T_1 _vertebral body and the lower end plate of T_12 _vertebral body. **D**_R _(**D**_L_) represents the distance from the midpoint of the distal end-plate of each vertebra body (T_1–12_) to the outline of the lateral border of the right (left) thoracic cage. These distances are standardised by dividing by the measured T_1 _– T_12 _distance (H). They are termed segmental right and left thoracic ratios TRs. Ratios are also calculated segmentally for the total width of the chest (right plus left measured lengths), [12].

On each chest radiograph, the outline of the lateral border of the thorax is drawn, Figure 1. Next, the mid-point of the distal end-plate at each vertebral body from T1–12 is marked. Then at each segment, the distance from the middle of the end-plate to each outline of the right and left thoracic cage is measured. These distances are standardized by dividing by the measured T1–12 distance. They are termed segmental left and right *Thoracic Ratios *(TRs). Ratios are also calculated segmentally for the total width of the chest (left plus right measured lengths) [11]. In a small number of the chest radiographs the lower two ribs were difficult to be defined, but in the films selected there were often abdominal and occasionally spinal radiographs to facilitate the measurements. No such problems were found for the spinal radiographs.

### Radiological measurements on the scoliotics' rib-cage

**Figure 2:**
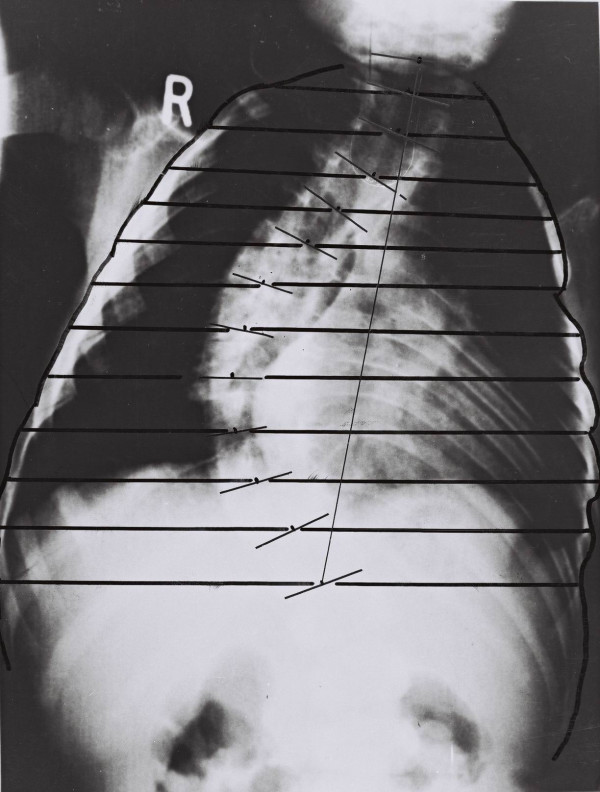
The method of thoracic ratios in the scoliotic rib – cage of a boy aged 1 year 7 months.

On PA spinal radiographs the outline of the lateral border of the thorax was drawn, Figure 2. The method used to calculate the TRs was similar to that used on the chest radiographs with the exception that the T1–12 distance was corrected for loss of height using the formula of Hodgett et al [14], log y = 0,683 + 0,014x, where y = calculated trunk height loss in mm and x = Cobb angle). They are termed segmental convex and concave TRs.

At the time of the initial examination, the Cobb angle was measured. [15].

Vertebral rotation was measured segmentally using the template of Perdriolle, [16] including apical vertebral rotation.

Vertebral tilt was measured segmentally as the angle, which the lower border at each vertebra (T1–L5) forms with a transverse line drawn at right angles to a vertical line from S1 [17]. Vertebral tilt angles below the transverse line are deemed positive and above negative.

### Scoliotic patients

The PA spinal radiographs of 24 children with progressive IIS were evaluated using the methods described previously.

In this group, there were 16 boys and 8 girls. Their mean age was 4.1 years old (range 1.1–9.1, SD 2.7). The mean Cobb angle was 54° (range 32°–78°). None of the patients had received surgical treatment. A few of the patients had been treated with plaster casts and/or brace. Their average age at onset was 2 years; the average interval between onset and the radiological evaluation was 34 months.

During follow up, the 24 patients received different treatments, namely, Group I: combined anterior and posterior surgery leading to apical vertebral derotation (n = 9), [2]; GroupII: posterior rodding alone (n = 5), [2,3] and Group III: conservative treatment with brace and/or plaster (n = 10) [3].

At follow-up, apical vertebral rotation was measured for each of the 24 patients. The average period of follow-up was 3.4 years in group I, 5.7 years in group II and 6.8 years in group III.

### Control subjects

Anteroposterior chest radiographs were obtained from 233 children aged 1–10 years attending the Accident and Emergency Department at the University Hospital, Nottingham during the 1989–90, Table 1. The children had minimal disorders or diseases involving trauma, infections, foreign bodies, heart murmurs and mild asthma. None of the patients had a scoliosis of 5 degrees or more. Two patients with congenital fusion of upper ribs both in a hemithorax were identified and shown here. Radiographs which were oblique were excluded. The chest radiographs were usually obtained in full inspiration. The age of each subject was calculated as decimal age.

**Table 1:** Number of boys and girls of the control group by age (n = 233)

**AGE**	**BOYS (n = 105)**	**GIRLS (n = 128)**
1–1.999	13	18
2–2.999	11	19
3–3.999	16	21
4–4.999	11	12
5–5.999	12	13
6–6.999	10	12
7–7.999	12	10
8–8.999	8	12
9–9.999	12	11

Children younger than 1 year all were not included in the analysis. All the 233 control children (105 boys and 128 girls) aged 1–10 years were used in the comparison with the scoliotic patients. The mean age was 5,049 ± 2,592 (SD) years.

### Statistical analysis

The statistical techniques included, Mann-Whitney for non-paired nonparametric groups, for Thoracic ratios of the control group between boys and girls, see Table 2, and for Thoracic ratios of the control group and the scoliotics, see Table 3, Spearman correlation coefficient, to test the correlation of vertebral rotation early in management with the apical vertebral rotation at follow up, multiple linear regression analysis and ANOVA of the various studied parameters.

**Table 2:** Thoracic ratios (left plus right), of the control group according to spinal level and sex (boys n = 105, girls n = 128)

**Spinal Level**	**BOYS**		**GIRLS**		
	
	**Mean**	**± 1 SD**	**Mean**	**± 1 SD**	**P^x ^(boys/girls)**
1	0.526	0.075	0.535	0.079	NS
2	0.678	0.081	0.677	0.084	NS
3	0.777	0.083	0.775	0.088	NS
4	0.858	0.079	0.856	0.088	NS
5	0.920	0.081	0.913	0.088	NS
6	0.970	0.083	0.954	0.089	NS
7	1.005	0.085	0.984	0.087	NS
8	1.032	0.089	1.007	0.090	*
9	1.049	0.096	1.023	0.092	*
10	1.068	0.098	1.039	0.095	*
11	1.070	0.102	1.043	0.095	NS
12	1.053	0.107	1.022	0.099	NS

^x^Mann-Whitney Test

NS Non Significant

* Statistically significant

**Table 3:** Infantile idiopathic scoliosis compared with controls: Thoracic ratios (left plus right) according to spinal level (scoliosis n = 24, controls n = 233)

**Spinal Level**	**SCOLIOTICS**		**CONTROLS**		
	
	**Mean**	**± 1 SD**	**Mean**	**± 1 SD**	**P^x ^(IIS/controls)**
1	0.447	0.063	0.531	0.074	***
2	0.563	0.102	0.677	0.082	***
3	0.667	0.084	0.776	0.085	***
4	0.738	0.092	0.857	0.084	***
5	0.602	0.097	0.916	0.085	***
6	0.850	0.100	0.961	0.086	***
7	0.890	0.106	0.994	0.087	***
8	0.919	0.105	1.018	0.090	***
9	0.921	0.143	1.035	0.094	***
10	0.958	0.118	1.052	0.097	***
11	0.976	0.122	1.055	0.099	***
12	0.849	0.166	1.036	0.104	***

^x^Mann-Whitney Test

*** Statistically significant

## Results

### Thoracic ratios in control subjects

**Figure 3:**
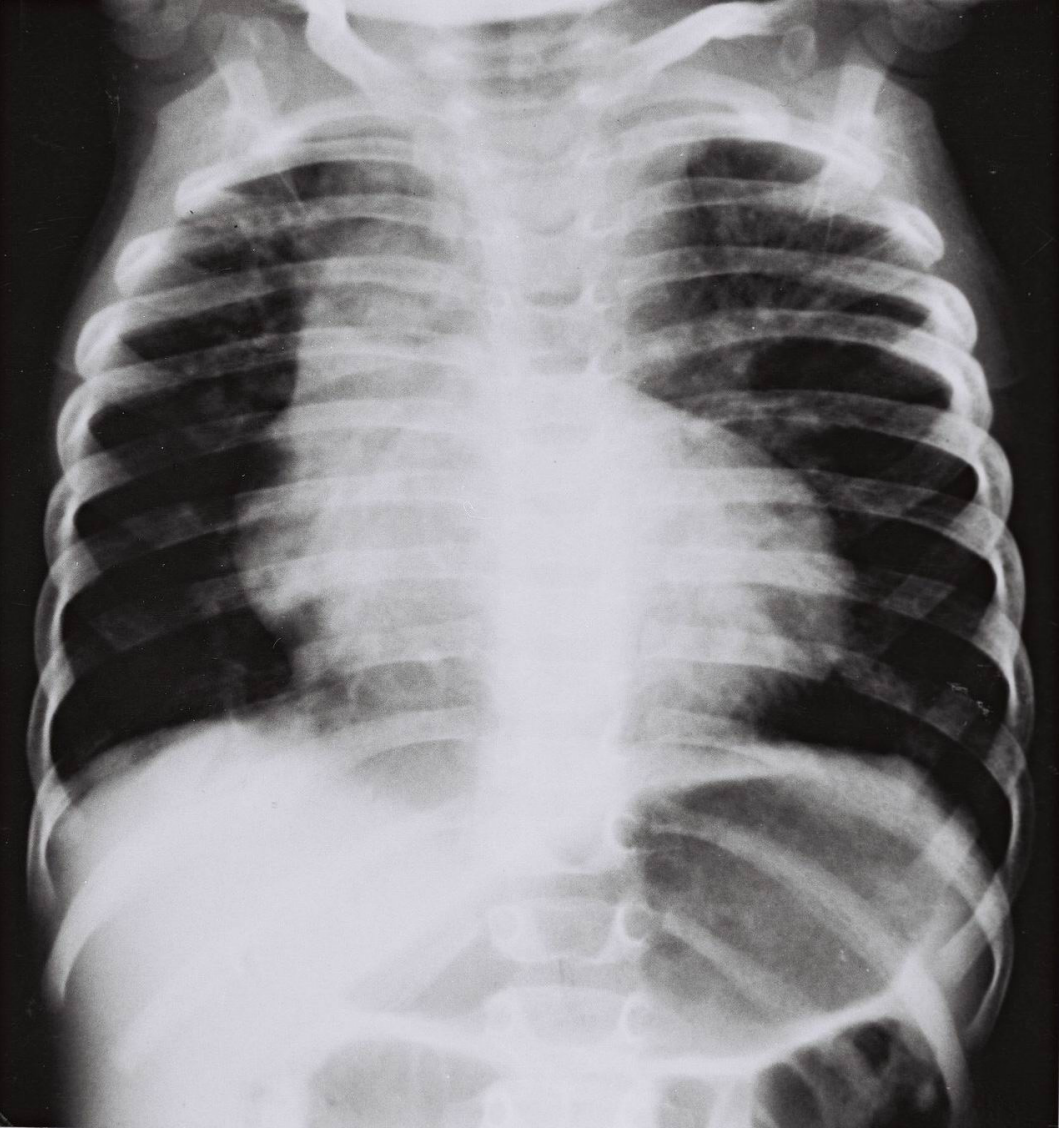
Chest radiograph of a normal girl aged 18 months, showing the difference of the shape of the thorax compared with scoliotic one (see figure 2).

Figure 3 shows a chest radiograph for a girl aged 18 months.

Table 2 shows the thoracic ratios (left plus right) by level and sex. It is seen that there is no statistically significant difference between boys and girls at T1–7 and at T11 and T12 level. There is a marginally statistical significant sex difference at T8–10 with the girls having narrower chests than boys at these levels (p ≈ 0.05, not p < 0.05). In view of these findings, the thoracic ratios are pooled for control boys and girls in Table 3. The comparison of the scoliotics with the controls is therefore practically valid for T1–12.

### Thoracic ratios in scoliotic patients

**Figure 4:**
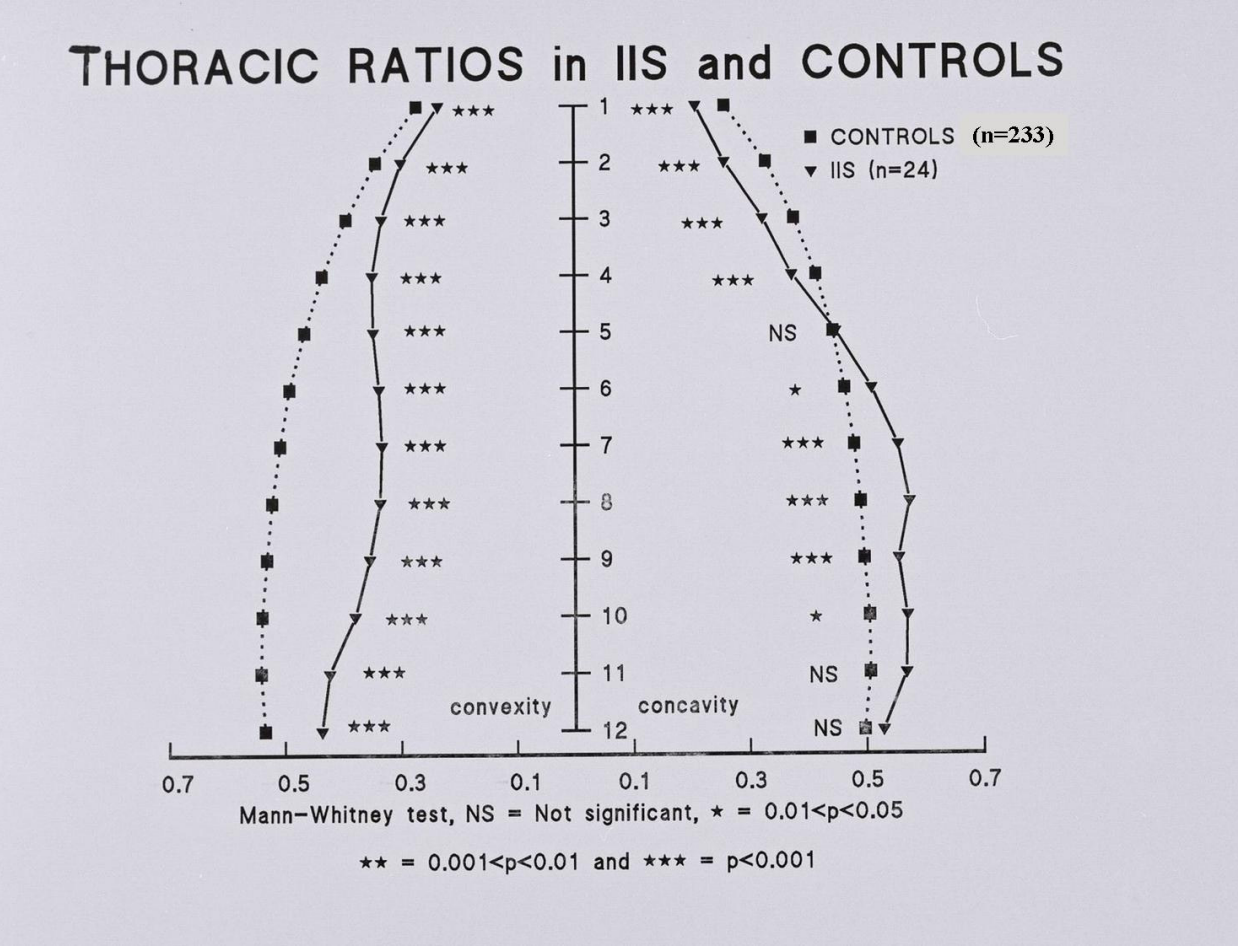
Thoracic ratios for the sample of patients with IIS compared with controls. The scoliotic thorax is significantly narrower than that of the controls at all spinal levels.

Figure 2 shows a spinal radiograph of a boy aged 1 year 7 months with progressive IIS. Figure 4 and Table 3 show the thoracic ratios for the sample of patients with IIS compared with controls. The comparison shows that the scoliotic thorax is significantly narrower than that of the controls at all spinal levels.

### Segmental vertebral rotation

**Figure 5:**
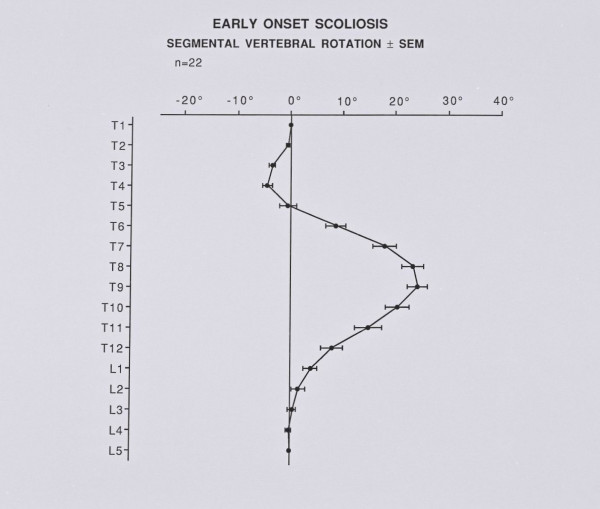
Segmental vertebral rotation at the initial examination of IIS patients.

Figure 5 shows a plot of segmental vertebral rotation at the initial examination.

### Segmental vertebral tilt (frontal plane)

**Figure 6:**
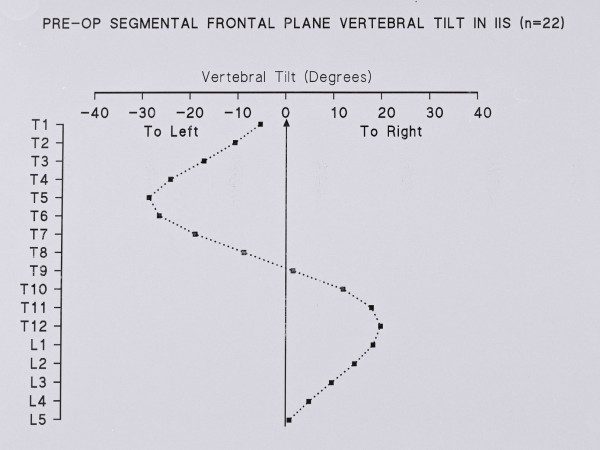
Segmental vertebral tilt at the initial examination of IIS patients.

Figure 6 shows a plot of segmental vertebral tilt in the frontal plane (PA radiograph) at the initial examination.

### Counter – rotation at T4 in relation to apical vertebral rotation follow – up

**Figure 7:**
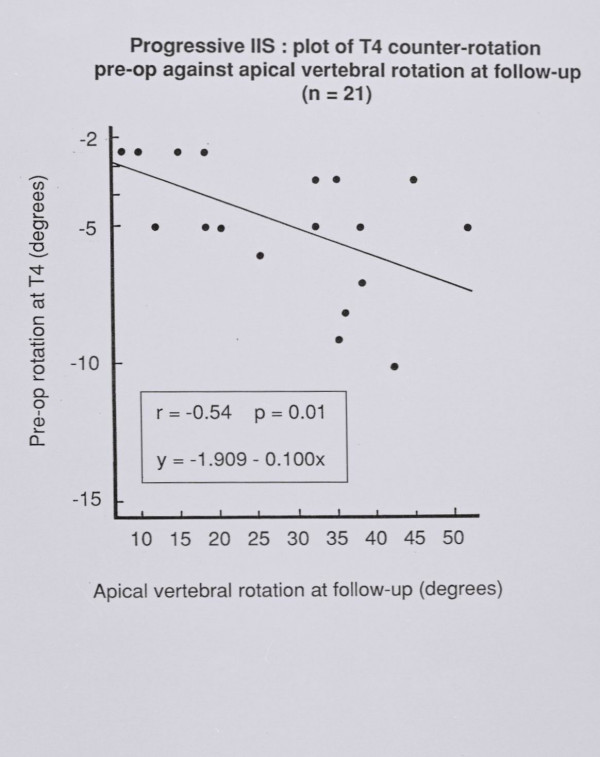
Progressive IIS: plot of T4 counter – rotation pre-op against vertebral rotation at follow-up (n = 21).

Figure 7 shows that the vertebral rotation at T4 early in management correlates significantly with the apical vertebral rotation at follow up. This association is not found for other upper thoracic levels. Nor is an association found between the initial T4 rotation and apical vertebral rotation preoperatively or postoperatively.

### Chest radiographs with rib – fusions

**Figure 8:**
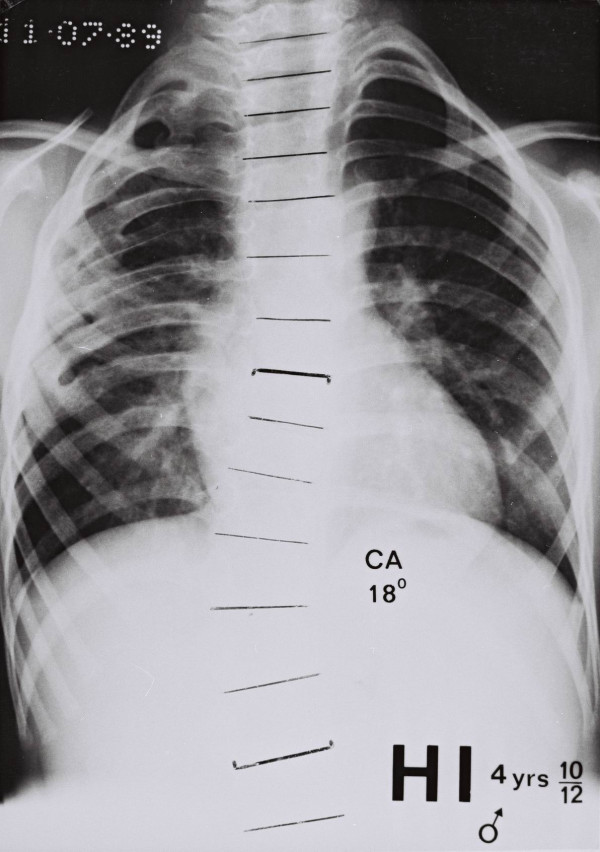
Patients with congenital rib abnormalities and scoliosis.

**Figure 9:**
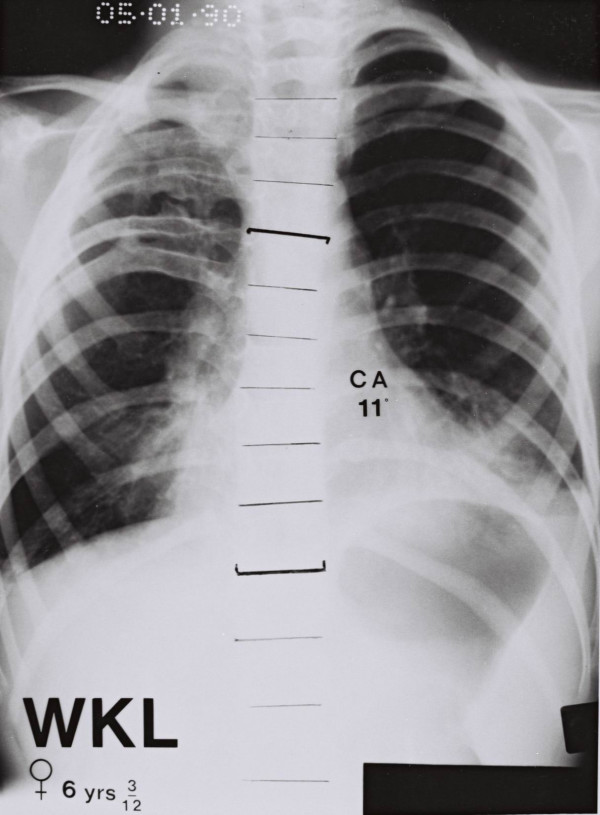
Patients with congenital rib abnormalities and scoliosis.

Figure 8 and figure 9 show the two patients in the series with congenital rib abnormalities. In the boy aged 4 years (Figure 8), the defect involves the upper right hemithorax as low as the eighth rib. There is a scoliosis convex to the right below the last level of the rib fusions (Cobb angle 18°, curve limits T8–L1). In the girl aged 6 years (Figure 9), the defect involves the right upper hemithorax as low as the sixth rib. There is a scoliosis convex to the right below the last level at the rib fusions (Cobb angle 11°, curve limits T6–11). In both the patients the affected upper right hemithorax is narrower than that on the left.

## Discussion

### General

This study considers only frontal plane radiographs. Currently more frequently three-dimensional analysis is used as a procedure to study the morphology of scoliotic curvatures as any study based exclusively on coronal plane has its limitations. Dansereau el al. [4] proposed a 3-D rib cage assessment, which certainly offers interesting possibilities but requires special equipment. Nevertheless from the practical point of view the studies based on anterior-posterior (a-p) radiographs may assume a valuable contribution. The most important radiological parameters are designed and measured on a-p radiographs (Cobb, Mehta, Perdriolle angles). Lateral radiographs are not systematically made to children with scoliosis. In the majority of hospitals the material accessible for retrospective studies contains almost exclusively frontal plane radiographs.

### The method

The TRs method expresses the width of the left and right hemithoraces segmentally in relation to the distance T1–12. In the chest radiographs of patients with minor disorders and diseases and straight spines, we have discussed elsewhere that three major factors determine the TRs: a) rib length, b) thoracic spinal length and c) the obliquity of the ribs measured as rib – vertebra angles [18]. In patients with idiopathic scoliosis additional factors include:

a) The lateral curvature which reduces the T1–12 distance which we correct using a formula [14].

b) The rotation of the vertebra in relation to the sagittal plane is maximum at the apex of the spinal curve; so that the mid – points of the vertebrae on the PA radiograph are not comparable to the mid – points of vertebrae in a straight spine.

c) The lateral spinal curvature is associated with the spinal displacement towards the convexity which narrows the convex hemithorax and broadens the concave hemithorax. This spinal displacement is associated with deformed convex ribs and the rib hump.

Due to the complexity of assessing TRs separately for the left and right hemithorax of scoliotics, we use combined ratios for purposes of comparison, Table 3. Figure 4 provides a general indication of the comparison between each hemithorax of the scoliotics and controls.

In the upper chest, the vertebral tilt decreases from T5 - T1, Figure 6. The counter – rotation in the upper thoracic spine is maximal at T4, Figure 7. Hence, the comparison of scoliotics with controls again needs combined ratios because of the problem of identifying the mid – point of the vertebrae.

### The funnel – shape of the upper chest in IIS

The narrow rib – cage at T1–4 in children with IIS compared with the controls is very highly significant, Table 3. The funnel – shaped upper thoracic cage of IIS is like that of each of: a) a normal human foetus, b) asphyxiating thoracic dysplasia (Jeune's disease) [19] and c) a normal adult rabbit.

The finding suggests that there are different patterns of postnatal growth in the upper and lower ribs of the human [18].

### Rotation of T4 in relation to apical vertebral rotation at follow up – thoracogenic scoliosis

Figure 7 shows that the counter – rotation of T4 predicts the apical vertebral rotation at follow – up (r^2 ^= 0.29). Hence 29% of the apical vertebral rotation at follow up is explained by the counter – rotation initially present at T4. By the time of follow – up, various treatments had been used namely, combined anterior and posterior surgery, posterior rodding alone and conservative treatment. We conclude that treatment is not the only factor contributing to apical vertebral rotation at follow – up. Our findings provide support for the conclusion of Perdriolle and his colleagues that 'specific rotation' (the sum of the two angle of rotation measured on the two vertebrae adjacent to the upper end – vertebra) can be used prognostically in IIS [16,20-22].

### Is the funnel – shaped chest of progressive IIS related to the predictive value of counter – rotation at T4? – a hypothesis

The hypothesis suggested is that the predictive value of vertebral rotation at upper limit of a thoracic curve of IIS reflects impaired rib control of spinal rotation due to neuromuscular factors; the latter also contributes to development delay of upper ribs leading to the funnel – shaped chest of progressive IIS. This hypothesis explains an observation of Bisgard [23] in connection with thoracogenic scoliosis: with each successive stage of multistage thoracoplasties, the apex of the curve correspondingly moves downward.

### Congenital rib fusions of one upper hemithorax (hemi – funneling)

Figures 8 and 9 show that congenital rib fusions in an upper hemithorax can be associated with a) narrowing of the hemithorax on the affected side and b) a scoliosis curve below the level (hemi – funneling) of rib – fusions which is convex to the side of the rib defect. These findings have relevance to a theory of aetiology for idiopathic scoliosis [24-27].

### Impact on the latest thoughts on scoliosis aetiology

Sevastic et al indicated that the normal thoracic spine is maintained in a state of equilibrium by a balance of the opposing force from the ribs and their surrounding structures and that imbalance of these mechanical forces predisposes for development of spinal deformities. [28].

The funnel-shaped upper chest could be considered an early expression of rib cage asymmetry and seems to unbalance the symmetric forces from the ribs that act on the spine and may lead to the development of the spinal deformity.

Furthermore a) the IIS thorax being narrower than that of the controls, b) the IIS upper chest being funnel-shaped and c) the vertebral rotation at the upper limit of the thoracic curve of IIS having a predictive value on follow up after treatment are facts which may well be considered as reflections of impaired rib control of spinal rotation possibly due to neuromuscular factors which contribute also to the funnel-shaped chest.

## Authors' contributions

TBG was the principal investigator of the study, conceived the idea of thoracic ratio's method, conducted the collection of data, performed the statistical analysis, created the graphs and the images and involved in drafting the article. GRB supervised the work, involved in drafting the article and participated in the whole project of the study. ESV helped in manuscript drafting and in the interpretation of data. JKW provided the patients, supported the project and made constructive comments on the article. All the authors read and approved the final manuscript.
